# Computerized Psychological Interventions in Veterans and Service Members: Systematic Review of Randomized Controlled Trials

**DOI:** 10.2196/30065

**Published:** 2022-06-03

**Authors:** Rahel Pearson, Emily Carl, Suzannah K Creech

**Affiliations:** 1 Veterans Health Administration Veterans Integrated Service Network 17 Center of Excellence for Research on Returning War Veterans Central Texas Veterans Affairs Healthcare System Waco, TX United States; 2 Department of Psychology University of Texas Austin, TX United States; 3 Department of Psychiatry and Behavioral Sciences Dell Medical School of the University of Texas Austin, TX United States

**Keywords:** computer, digital, internet, interventions, veterans, service members, review, mobile phone

## Abstract

**Background:**

Computerized psychological interventions can overcome logistical and psychosocial barriers to the use of mental health care in the Veterans Affairs and Department of Defense settings.

**Objective:**

In this systematic review, we aim to outline the existing literature, with the goal of describing: the scope and quality of the available literature, intervention characteristics, study methods, study efficacy, and study limitations and potential directions for future research.

**Methods:**

Systematic searches of two databases (PsycINFO and PubMed) using PRISMA (Preferred Reporting Item for Systematic Reviews and Meta-Analyses) guidelines were conducted from inception until November 15, 2020. The following inclusion criteria were used: the study was published in an English language peer-reviewed journal, participants were randomly allocated to a computerized psychological intervention or a control group (non–computerized psychological intervention active treatment or nonactive control group), an intervention in at least one treatment arm was primarily delivered through the computer or internet with or without additional support, participants were veterans or service members, and the study used validated measures to examine the effect of treatment on psychological outcomes.

**Results:**

This review included 23 studies that met the predefined inclusion criteria. Most studies were at a high risk of bias. Targeted outcomes, participant characteristics, type of support delivered, adherence, and participant satisfaction were described. Most of the examined interventions (19/24, 79%) yielded positive results. Study limitations included participant characteristics limiting study inference, high rates of attrition, and an overreliance on self-reported outcomes.

**Conclusions:**

Relatively few high-quality studies were identified, and more rigorous investigations are needed. Several recommendations for future research are discussed, including the adoption of methods that minimize attrition, optimize use, and allow for personalization of treatment.

## Introduction

Most individuals with diagnosable mental health disorders do not have access to adequate care [1]. Computerized psychological interventions are well-positioned to address this treatment gap, as these interventions provide a cost-effective and easily accessible alternative to traditional face-to-face mental health care [2,3]. Computerized psychological interventions, often delivered through the internet, have grown steadily in popularity over the past decade. Computerized psychological interventions may be effective for the treatment of a range of mental health disorders. Meta-analyses have supported the efficacy of computerized psychological interventions in the treatment of depression [4-6], anxiety [4,7], posttraumatic stress disorder (PTSD; [8]), substance use disorders (SUDs; [9,10]), and insomnia [11], with effect sizes ranging from small [5,7,9] to medium-large [4,6,11]. Various meta-analyses have outlined the therapeutic benefit of specific computer-delivered treatment modalities, such as cognitive behavioral therapy (CBT) [4,11], acceptance and commitment therapy [12], and mindfulness-based therapy [13]. There is evidence that computerized psychological interventions are as effective as face-to-face interventions [4,14], suggesting that a variety of mental health conditions can be addressed by computerized treatments with the potential for widespread dissemination of such therapies.

As the body of evidence supporting computerized psychological interventions is robust, it is not surprising that the Department of Veterans Affairs (VA) and the Department of Defense have increasingly implemented these interventions [15], with increasing studies examining computerized psychological interventions in current and former service members. To our knowledge, there has been no systematic review of the literature examining computerized psychological interventions in veterans and service members. Reviews of computerized psychological interventions in general community samples might not generalize to veterans and service members, given the unique considerations in terms of gender ratio, severity of comorbid conditions, socioeconomic factors, and situational or environmental exposures. The use of computerized psychological interventions in veterans and service members will likely continue to rise. The objective of this systematic review is to examine the literature on randomized controlled trials (RCTs) using computerized psychological interventions in veteran and military populations by describing study characteristics and summarizing the efficacy of these interventions. We also aim to examine the quality and limitations of the identified studies. Finally, recommendations for future research will be made.

## Methods

### Study Selection and Data Collection

**Figure 1:**
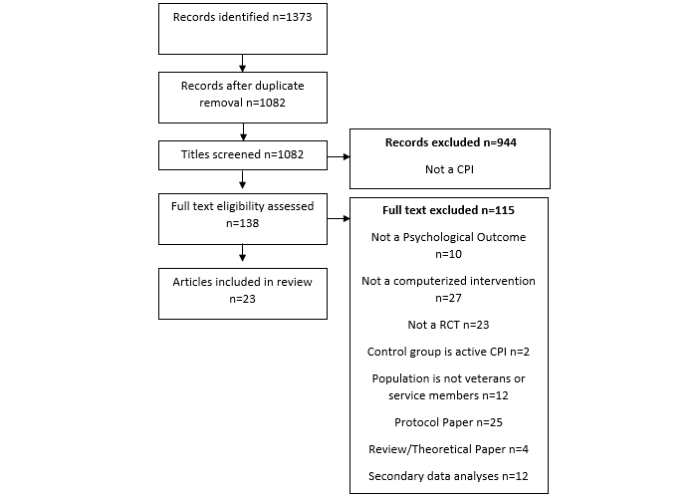
Flow chart of records identified through database screening.

Systematic searches of two databases (PsycINFO and PubMed) were conducted from inception until November 15, 2020. The search terms are described in Multimedia Appendix 1 (adapted from Moore et al [16]). Duplicates were removed, and the reference lists of the included studies were examined for additional articles. A final list of included studies was circulated among colleagues with subject matter expertise to verify that no relevant papers were omitted. The following inclusion criteria were used: (1) the study was published in an English language peer-reviewed journal, (2) participants were randomly allocated to a computerized psychological intervention or a control group (non–computerized psychological intervention active treatment or nonactive control group), (3) an intervention in at least one treatment arm was primarily delivered through the computer or internet with or without additional support, (4) participants were military veterans or service members, and (5) the study used validated measures to examine the effect of treatment on psychological outcomes. Validated psychological outcome measures are those measures with demonstrated reliability and validity that quantify mood, well-being, emotion, affect, and/or psychosocial functioning. The outcomes examined were mental health disorders, as defined by the Diagnostic Statistical Manual, fifth edition (eg, SUDs and neurocognitive disorders), as well as psychosocial and behavioral correlates of these disorders (eg, romantic relationship dysfunction and anger). As commonly used VA smartphone apps are designed to be used in conjunction with traditional face-to-face treatment [17], they were not included. Papers were reviewed for inclusion at the title, abstract, and full paper levels. RP determined if studies met the inclusion criteria, and any ambiguity was discussed with SC until a final decision was reached. Using a standardized form, intervention characteristics, population characteristics, study design, methods, procedures, and outcomes were recorded. The number of papers identified, screened, and included is reported in Figure 1.

### Data Synthesis

Findings were grouped by the targeted psychological outcome and population examined (veterans and/or service members). These subgroups used similar methods, such as intervention techniques and recruitment tactics, allowing for a more direct comparison. Study outcomes were examined and visually presented in a harvest plot by these subgroups, as well as the control group (active or inactive), to provide a more nuanced understanding of the study results. If a study included both an active and inactive control group, the computerized psychological intervention was compared with the active control group to provide a more robust test of computerized psychological intervention efficacy. Overall, the studies were heterogeneous, and subgroups of similar studies were very small (≤3), making meta-analysis inappropriate. A narrative synthesis of the effects was conducted to describe the efficacy of interventions on primary outcome measures. Effect sizes (Cohen *d* of Hedges *g* if n<20) were extracted or calculated where possible. The authors of the included studies were contacted when insufficient data were provided in the article.

### Quality Assessment

The Cochrane risk of bias tool [18] was used to determine the methodological quality of the included studies. The assessed features were sequence generation and allocation sequence concealment (selection bias), blinding of personnel (performance bias), blinding of outcome assessment (detection bias), incomplete outcome data (attrition bias), and selective outcome reporting (reporting bias). Two authors (RP and EC) independently rated the risk of bias as *high risk* (eg, sequence generation: randomization based on birthday), *low risk* (eg, sequence generation: block randomization with randomly varied blocks sizes), or *unclear risk* (eg, sequence generation: no information on randomization method). Overall bias was rated as *low* if ratings of bias were low in all domains or if bias was *unclear* in 1 domain, and this was unlikely to have biased the study outcome. Overall bias was rated as *moderate* if bias was high in 1 domain or *unclear* in 2 domains, and this was unlikely to have biased the study outcome. Overall bias was rated as *high* if bias was rated as *high* in 1 domain or *unclear* in 2 domains, and this was likely to have biased study outcomes. Similarly, overall bias was rated as *high* if bias was rated as *unclear* in ≥3 domains or *high* in ≥2 domains. Ratings were compared, and the 2 raters discussed discrepancies until a consensus was reached.

## Results

### Intervention Characteristics

#### Overview

Characteristics of the included studies are outlined in Multimedia Appendix 2 [19-42]. Most computerized psychological interventions targeted PTSD and SUDs. Most computerized psychological interventions were web-based, although some studies used software programs installed on study computers [19-23]. Intervention content was often presented with graphics and interactive features that allowed tailored treatment content based on participant characteristics [19,24-32]. Computerized psychological interventions offered varying degrees of guidance in the 23 studies: 3 (13%) studies [24,29,33] provided no human contact during any study phase, 15 (65%) studies [19-23,25-27,31,34-39] provided administrative contact (eg, reminders, use support, and structured or semistructured assessment), and 5 (22%) studies [28,30,32,40,41] included therapeutic contact.

#### PTSD Interventions

Of the 24 computerized psychological interventions, 8 (33%) addressed PTSD and related symptoms. Of the 23 studies, 3 (13%) studies examined expressive writing interventions of different lengths [38-40] with PTSD symptomatology as the primary outcome, 3 (13%) studies [26,28,36] tested the efficacy of computerized psychological interventions that included a variety of CBT-based techniques, such as cognitive restructuring and behavioral modification. McLean et al [32] computerized prolonged exposure, an evidence-based treatment (EBT) for PTSD, and Larsen et al [37] examined the efficacy of cognitive training on PTSD symptoms.

#### SUD Interventions

Interventions addressing SUDs were primarily focused on drinking problems (3/23, 13% studies; 4/24, 17% interventions) [22,29,33] or drinking or substance use problems and PTSD [24,35]. One of the studies [25] tested the efficacy of a smoking cessation intervention. Interventions addressing only drinking problems were completed in one sitting [22,29,33]. All interventions included alcohol assessment feedback and psychoeducation on alcohol use. In addition, interventions presented peer-specific norms for alcohol use [22,29,33] and included motivational techniques [22,29]. Interventions addressing comorbid alcohol/substance use and PTSD were CBT based and significantly longer than interventions addressing alcohol use in isolation [24,35]. Both interventions integrated CBT components and motivational techniques aimed at trauma and substance or alcohol use. Acosta et al [35] also offered optional trauma exposure modules, although there was limited engagement with this content. Calhoun et al [25] tested the efficacy of a web-based smoking intervention, Quitnet, which included behavioral goal setting and social support components.

#### Other Interventions

Approximately 8% (2/24) of interventions targeted depression: Bedford et al [19] examined a 6-session problem-solving intervention, whereas Pfeiffer et al [30] examined Beating the Blues, a well-established CBT for depression intervention. CBT interventions were also used to treat anger [21] and insomnia [31] and decrease suicidality by targeting perceived burdensomeness and thwarted belongingness [23]. Cooper et al [20] examined a brain fitness program to ameliorate the deleterious effects of mild traumatic brain injury. Finally, 2 interventions included participants and their partners. Kahn et al [27] examined an intervention aimed at promoting postdeployment rehabilitation, which included mindfulness-based techniques and massage therapy instructions. Salivar et al [41] tested 2 interventions, combined for data analyses, for low-income couples who experienced relationship distress. Interventions integrated conflict management with improving communication, commitment, and positivity (intervention 1) or acceptance and implementing behavioral change (intervention 2).

### Recruitment and Sample Size

A subset of trials recruited veterans from one [21,25,37,40] or more [22,30,34-36,38] VA facilities. Approximately 13% (3/23) of trials [22,30,35] contacted veterans who were likely to meet the study inclusion criteria based on information obtained from the VA electronic medical record system, and another sent out eligibility questionnaires and US $5 incentives to a large (N=15,686) random sample of veterans [39]. Several trials [19,24,26,27,33,34] recruited participants through targeted emails on the web (eg, emails sent out to members of veterans’ associations) or through Facebook and other social media advertising. In addition, veterans were recruited from university campuses [19,34,37] and the community [23,34,37]. Approximately 30% (7/23) of studies recruited active duty personnel [20,28,29,31] from military installations and Department of Defense sites or a combination of active duty personnel and veterans [32,36,41]. Of the 23 studies, 4 (17%) studies included all or majority US army personnel [20,31,32,36], 1 (4%) study recruited from various military branches at different installations and sites [29], and 2 (9%) studies did not specify the military branches represented in their sample [28,41]. Pemberton et al [29] recruited a convenience sample at 8 military installations and was able to recruit the largest sample size (N=3070, compared with a sample range of N=40 to N=180 for other studies recruiting service members). However, the study authors noted several sample limitations, such as the low prevalence of drinking problems and high study attrition.

Web-only trials that recruited nationally through social media or by US mail were generally more successful in obtaining large sample sizes [26,27,33,38,39]. Sample sizes were generally reduced in pilot trials [34,37,38], if ≥1 study arm required intensive participant contact (eg, face-to-face treatment and magnetic resonance imaging [21,31,34]), or if eligibility criteria were stringent and/or required specialty assessment [20].

### Adherence and Attrition

Adherence to the intervention was addressed in 87% (20/23) of examined studies, although only 9% (2/23) of studies clearly defined the level of engagement that differentiated adherence versus nonadherence (ie, >80% of sessions completed [37] or >5 sessions completed [30]). The remaining studies provided a quantitative assessment of participant engagement with the intervention, such as the time spent or sessions completed. Adherence, defined as completion of all study sessions, ranged from 25% to 100% (based on 12/23, 52% of studies with available data [19,22,24,29,32-36,38,40,41]).

Attrition, defined as a loss to follow-up, was reported in all studies. Across all studies, 36.25% (2994/8260) of participants were lost at the first follow-up time point, which often coincided with the posttreatment assessment. At the second follow-up time point (average follow-up length was 16 weeks), 45.59% (3542/7770) participants across 87% (20/23) of studies were lost. When computerized psychological intervention attrition at the first follow-up time point was examined by support type, attrition was 51.78% (2311/4463) in the no support group (3/23, 13% studies; 4/24, 17% interventions), 17.29% (548/3170) in the administrative support group (15/23, 65% studies), and 21.5% (135/629) in the therapeutic support group (4/23, 17% studies).

### Risk of Bias

#### Overview

The Cochrane risk of bias tool [18] assessment of bias consensus ratings for the included studies is found in Table 1.

**Table 1:** Consensus ratings for Cochrane assessment of bias.

Study	Random sequence generation	Allocation concealment	Blind outcome assessment	Incomplete data	Selective reporting	Overall risk of bias
Acosta et al [35]	Unclear	Unclear	Low	Low	Low	Moderate
Bedford et al [19]	Low	Low	Low	High	Unclear	High
Brief et al [24]	Unclear	Unclear	Low	High	Unclear	High
Calhoun et al [25]	Unclear	Low	Low	Unclear	Unclear	High
Clausen et al [34]	High	High	Low	High	Low	High
Cooper et al [20]	Unclear	Unclear	Low	High	Low	High
Cucciare et al [22]	Low	Unclear	Low	Low	Low	Low
Engel et al [36]	Low	Low	Low	Low	Unclear	Low
Hobfoll et al [26]	Low	Unclear	Low	High	Unclear	High
Kahn et al [27]	Low	Unclear	Low	Low	Low	Low
Krupnick et al [40]	Unclear	Unclear	Low	High	Unclear	High
Larsen et al [37]	Unclear	Unclear	Low	Unclear	Low	High
Litz et al [28]	Unclear	Unclear	Low	High	Unclear	High
McLean et al [32,42]	Unclear	Unclear	Low	High	Unclear	High
Pedersen et al [33]	Unclear	Unclear	Low	Low	Low	Moderate
Pemberton et al [29]	High	High	Low	High	Unclear	High
Pfeiffer et al [30]	Low	Unclear	Low	Unclear	Unclear	High
Possemato et al [38]	Unclear	Unclear	Low	High	Unclear	High
Salivar et al [41]	Unclear	Unclear	Low	Unclear	Unclear	High
Sayer et al [39]	Low	Unclear	Low	Low	Unclear	Moderate
Short et al [23]	Low	Unclear	Low	High	High	High
Taylor et al [31]	High	High	Low	Low	Low	High
Timmons et al [21]	Unclear	Unclear	Low	Unclear	Unclear	High

#### Sequence Generation

Of the 23 studies, 11 (48%) provided a detailed description of their process of sequence generation, 8 (25%) were rated as low risk for bias, and 3 (13%) were rated as high risk for bias. Studies that were at high risk for bias either discontinued randomization during the study because of technical difficulties [31] or an investigator-moving institution [34]. Pemberton et al [29] were unable to randomize as intended as internet speed limited the availability of interventions at certain study locations. Approximately 52% (12/23) of studies did not provide sufficient detail on their process of sequence generation (eg, specified that block randomization was used but failed to outline the process of selecting block size), and their risk of bias was rated as unclear.

#### Allocation Concealment

Of the 23 studies, 3 (13%) were rated as having a low risk for bias, 2 (9%) explicitly stated that study staff were blind to treatment allocation [19,25], and 1 (4%) used variable block size, blinding study staff to treatment allocation [36]. The 13% (3/23) of studies that were unable to randomize as intended (see *Sequence Generation* section; Taylor et al [29], Clausen et al [31], and Pemberton et al [34]) presumably had to unblind study staff to treatment allocation and were rated as having a high risk of bias. The remaining studies did not provide sufficient details about allocation concealment and were rated as having an unclear risk of bias.

#### Blind Outcome Assessment

All but 17% (4/23) of studies used self-report or physiological measures exclusively to assess outcomes, and these studies were rated as low risk for bias. The studies that used clinician-guided or rated assessment measures [20,28,32,34] specified that raters were blind to the treatment arm and were thus rated as having a low risk of bias.

#### Incomplete Data

Of the 23 studies, 17 (74%) clearly defined treatment attrition and stated that they used intention-to-treat (ITT) analyses [19,20,22,24-31,35-40]; however, 2 (9%) studies did not include all randomized participants in ITT analyses [20,38] and 1 (4%) study failed to impute missing data [19]. In addition, 30% (7/23) of these studies [19,20,24,26,28,29,38] reported high attrition (>15% difference in missing data between treatment arms and/or >40% missing data overall [43]; as cited in Berge et al [44]), which introduces risk for bias regardless of the statistical methods used to attenuate this risk. The remaining studies either conducted completer analyses [21,23,34,42], had missingness that was not random [23,32,34], or failed to specify attrition rates by treatment arm [41].

#### Selective Reporting

Approximately 35% (8/23) of trials preregistered their studies [20,22,27,31,34,35,37], and 30% (7/23) reported preregistered outcomes and were rated as having a low risk of bias. One of the studies [23] reported outcomes significantly different from those that were preregistered, and this study was rated as having a high risk for bias. One of the studies published a protocol paper outlining their methods and intended analyses and was rated as having a low risk for bias [33]. The remaining studies were not preregistered, and the risk for bias was rated as unclear.

### Outcomes and Satisfaction

**Figure 2:**
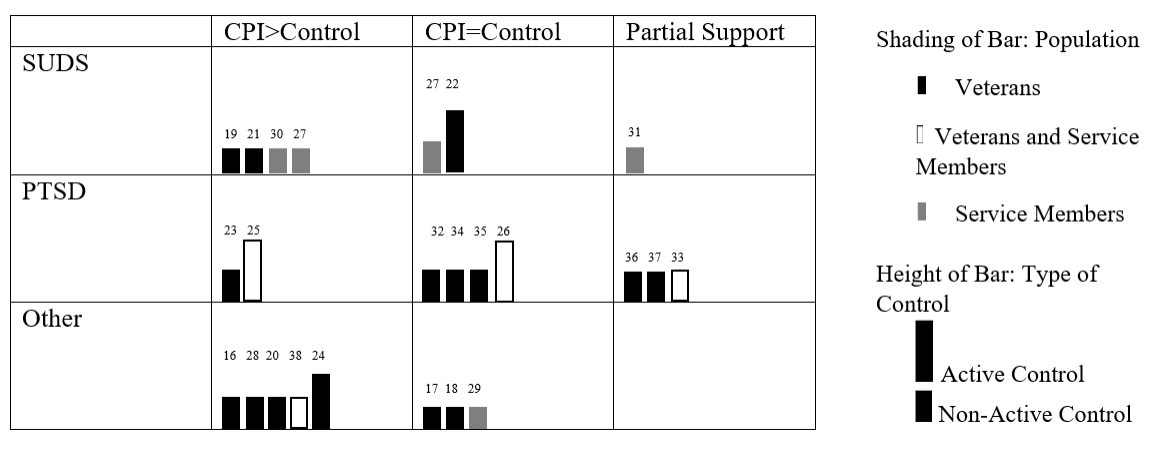
Harvest plot of study outcomes by intervention, population, and control group. PTSD: posttraumatic stress disorder; SUD: substance use disorder; TAU: treatment as usual.

Figure 2 presents the outcome data visually in a harvest plot, and study effect sizes are included in Multimedia Appendix 2.

Of the 23 studies, 13 (57%) reported positive results when comparing computerized psychological interventions to nonactive control groups or treatment as usual, 9 (39%) reported results that supported the specified hypotheses, and 4 (17%) reported partial support for the hypotheses. For example, Krupnick et al [40] reported that expressive writing (compared with treatment as usual) reduced PTSD hyperarousal symptoms; however, there was no significant movement in other PTSD domains. Sayer et al [39] found that expressive writing (compared with placebo writing) reduced physical complaints, anger, and general psychological distress but did not report a reduction in PTSD symptoms. Similarly, Acosta et al [35] and Engel et al [36] reported that gains in one domain (drinking and PTSD, respectively) failed to generalize to other outcomes.

Approximately 9% (2/23) of studies reported that the computerized psychological intervention under examination outperformed an active control treatment. Litz et al [28] found that self-management CBT (vs computerized supportive counseling) reduced daily measures of PTSD and depression, with 6-month follow-up reductions in depression, PTSD, and anxiety in the completer group. Kahn et al [27] found that their computerized psychological intervention led to improvements in various mental health–related outcomes when compared with residential treatment. Approximately 17% (4/23) of studies found that their computerized psychological intervention performed as well as the face-to-face treatment equivalent. Positive results found in computerized psychological interventions were comparable with those for in-person CBT for insomnia [31], in-person group anger inoculation training [21], therapist-led cognitive rehabilitation [20], and clinic-based smoking cessation care [25]. McLean et al [32] found that computerized prolonged exposure did not outperform non–trauma-focused face-to-face treatment when examining PTSD outcomes.

Approximately 17% (4/23) of studies failed to demonstrate significant treatment effects, with expressive writing [38], executive functioning training [34], and working memory training [37] for PTSD not outperforming placebo. Similarly, 50% (1/2) of the computerized psychological interventions for alcohol use examined by Pemberton et al [29] did not outperform the waitlist control group.

Satisfaction data were provided in several studies. Overall, participants felt that they benefited from computerized psychological intervention engagement: with 82% [35], 93% [38], 96% [41], and 76.1% [39] of participants noting positive treatment effects. One of the studies found similar satisfaction rates between the computerized psychological intervention and the in-person treatment equivalent [21]. Although participants generally reported high treatment satisfaction and acceptability, there were reports of computerized psychological interventions being difficult to complete [34], difficult to understand [35], or impersonal and time consuming [40].

## Discussion

### Principal Results and Comparison With Previous Work

We aimed to systematically review the literature examining computerized psychological interventions in veteran and military populations. Across 23 studies, 24 interventions met the inclusion criteria and were reviewed. PTSD and SUDs were the most commonly targeted clinical difficulties, and other outcomes included anger, depression, insomnia, traumatic brain injury, relationship distress, suicidality, and readjustment difficulties. Interventions spanned a range of modalities and mostly focused on veterans, although a subset of studies recruited active military personnel. Approximately 8% (2/24) of interventions included romantic partners, whereas the other interventions followed an individual format. Most studies provided administrative support only, 13% (3/23) of studies provided no support, and 22% (5/23) of studies provided clinically meaningful support. Results were mostly positive; however, only 13% (3/23) of studies reporting positive results were rated as low risk for bias. Similarly, all studies that did not report significant treatment effects were rated as having a high risk of bias. Therefore, we caution against interpreting these results as unambiguous evidence of clinical effectiveness or ineffectiveness. Although it appears that computerized psychological interventions hold promise for the treatment of psychological difficulties in veterans and military service members, there is a need for more high-quality evidence to increase the confidence with which conclusions can be drawn.

Given the broad inclusion criteria that allowed great heterogeneity in intervention content and outcomes, a limited number of RCTs were identified. This is especially true in comparison with the number of RCTs that examine computerized psychological interventions in the general population. For example, Andrews et al [4] identified 53 RCTs that targeted depression and anxiety. Although the literature search returned a substantial number of pilot trials and process papers, limited RCTs were identified, indicating that the step from examining feasibility to establishing efficacy has not been decisively made. Furthermore, a high or unclear risk of bias across multiple features was common, with attrition bias being a concern for 48% (11/23) of the studies reviewed, greatly reducing the confidence with which inferences can be made. There are generally high rates of attrition in computerized psychological intervention trials [45], and veterans might be at a higher risk of attrition from treatment [46]. Although 74% (17/23) of the studies included in this review attempted to compensate for missing data by using ITT analyses, 30% (7/23) of studies had such a significant loss of data that high risk for bias was introduced regardless of the statistical methods used to attenuate this risk. Future research should attempt to reduce data loss by incorporating procedures associated with improved study retention. For example, clear study completion deadlines and prescheduled posttreatment assessments have been shown to reduce attrition [47], and adherence is improved when interventions are designed to include persuasive technology (ie, technology designed to include elements of social influence, such as praise, personalization, and social learning [48]). Therapeutic support (vs no support or administrative support) is also associated with improved retention in computerized psychological intervention trials [49], although it is not clear whether the benefits of including therapist contact outweigh the limitations placed on intervention scalability. Recently, evidence [50,51] has supported the efficacy of single-session web-based interventions, which can maximize recruitment while minimizing attrition. Of the studies reviewed here, Pedersen et al [33] reported low attrition rates and medium effect sizes for a single-session intervention administered in a help-seeking sample, demonstrating that these interventions can be successfully adapted for use in veterans.

Attrition is associated with insufficient statistical power, especially when the initial sample sizes are small, as was the case in many of the studies reviewed. Future research should anticipate high rates of attrition and set recruitment goals to ensure adequate statistical power for detecting treatment effects. In addition to dropout, low study use is another common concern in studies examining computerized psychological interventions. Notably, only 9% (2/23) of studies [30,37] defined study use (eg, minutes spent in the program or modules completed) as constituting adherence versus nonadherence. Similarly, only 30% (7/23) of studies described the relationship between use metrics and study outcomes. It is important to further consider these metrics in computerized psychological intervention research, as there is evidence that a dose–response relationship exists for computerized psychological interventions. For example, increases in modules completed [52] and more frequent use [53] are related to greater improvement at posttreatment. Examining study use and its relationship to outcome would also elucidate which intervention components are associated with change, allowing for the optimization of treatment.

Several studies have noted that sample characteristics limit the generalizability of the results. As is the case with much veteran and military research, samples tended to be mostly male. There is a need for computerized psychological interventions addressing the unique needs of female veterans, with recent evidence suggesting that these interventions are feasible, satisfactory, and potentially beneficial [54]. Male veterans who experienced military sexual trauma and transgender veterans are other subpopulations that might benefit from computerized psychological interventions because of high mental health disorder rates and numerous barriers to establishing care [55,56]. Further, some studies limited enrollment to post–9/11 war veterans. This cohort is younger and might be more computer literate than the overall veteran population; however, given that a substantial proportion of veterans served in VA are ages >65 years [57], it is important to investigate the utility of computerized psychological interventions for this population. Although evidence suggests that computerized psychological interventions can provide additional care options for rural patients [58], there is a need to establish ways of extending coverage to veterans who live in rural areas without adequate internet infrastructure or transportation.

Several studies noted sample heterogeneity within treatment arms as a limitation. Although limiting sample variability can enhance the interpretability of findings, it also constrains generalizability. Alternatively, given a robust sample size, statistical methods can be leveraged to identify characteristics within heterogeneous samples associated with beneficial and/or adverse computerized psychological intervention treatment effects [59]. This would allow a stepped care approach, where minimally invasive and cost-effective treatments such as computerized psychological interventions are initially offered to those who are most likely to benefit, and resource-intensive face-to-face treatments are reserved for veterans or service members who require a higher level of care. Optimizing service delivery by identifying subsets of veterans or military personnel who are well-suited for computerized psychological intervention treatment is an important avenue of research, and initial explorations in this area are being reported [60].

VA is increasingly focusing on offering EBTs for mental health disorders. Although various interventions reviewed here included evidence-based practices (eg, exposure and motivational techniques), only 13% (3/23) of studies digitalized an EBT [30-32]. There is evidence that face-to-face EBTs used at VA can be successfully offered in a computerized format, as there have been positive trials of web-based exposure-based trauma treatment [61] and CBT for chronic pain [62] in general community samples. Similarly, there is an evidence base for web-based CBT [63] and acceptance and commitment therapy [64] for depression. Future research should continue to focus on digitalizing those treatments that have proven efficacy.

### Limitations

This review has several limitations. First, owing to small sample sizes and high rates of potential bias, conclusions that computerized psychological interventions are potentially beneficial for veterans and service members are tentative. Lack of reporting was common across studies, resulting in many *unclear* bias ratings, which introduces uncertainty in our overall assessment of bias. Second, findings might not generalize to the broader veteran population, as many of the reviewed studies limited enrollment to post–9/11 veterans. Third, only English language articles were included, and all the studies reviewed were conducted in North America with US veterans or service members, and results cannot be extrapolated to other contexts. Fourth, only 1 author (RP) evaluated the results of the search; having an additional reviewer of these results would have strengthened the methodology. Finally, the methods of the studies included in this review are diverse, which in some instances complicates direct comparisons. The study heterogeneity also precluded conducting a meta-analysis, which would be the most robust way of assessing intervention efficacy. As the literature examining computerized psychological interventions in veterans and service members grows, efforts should be made to synthesize results by conducting a meta-analysis, which would provide evidence for intervention utility in this population.

### Conclusions

Computerized psychological interventions are uniquely positioned to optimize treatment access and use for service members and veterans. These interventions could be integrated into a stepped care framework and reduce the burden on the health care system while increasing engagement with mental health services in this vulnerable population. Despite increased research interest, significant work remains in the development and evaluation of computerized psychological interventions targeted at veterans and service members. Although initial outcomes suggest that computerized psychological interventions are potentially beneficial for this population, much of the available research is at high risk for bias and fails to fully incorporate known evidence-based practices. There is an opportunity to design treatments that minimize threats to internal validity (eg, attrition and limited engagement) by including strategies to increase user motivation and by distilling treatments to include the most active intervention components. As veterans and service members report complex mental health challenges, as well as perceived and actual barriers in obtaining adequate care, there is an acute need to address the limitations of the existing literature.

## Appendix

Multimedia Appendix 1 Search terms for review.

Multimedia Appendix 2 Characteristics of the studies included in the review.

## Abbreviations

CBT: cognitive behavioral therapy
EBT: evidence-based treatment
ITT: intention to treat
PTSD: posttraumatic stress disorder
RCT: randomized controlled trial
SUD: substance use disorder
VA: Veterans Affairs
