# Supplementary material for: Computerized Psychological Interventions in Veterans and Service Members: Systematic Review of Randomized Controlled Trials
Source: J Med Internet Res. 2022 Jun 3;24(6):e30065. doi: 10.2196/30065 (PMC9206197; doi:10.2196/30065)
Supplement: Multimedia Appendix 1 [file jmir_v24i6e30065_app1.docx]

Appendix 1. Search Terms for Review

| Term 1 | Term 2 | Term 3 |
| --- | --- | --- |
| veteran* OR military | computer assist* OR computer tailor* OR computer base* OR computer guide* OR computer administer* OR computerized OR internet assist* OR internet tailor* OR internet base* OR internet guide* OR web assist* OR web tailor* OR web base* OR web guide* OR interapy OR software base* OR software assist* OR interactiv* OR internet OR online OR computer | treatment OR therapy OR intervention |
