# Supplementary figures and images for: Computerized Psychological Interventions in Veterans and Service Members: Systematic Review of Randomized Controlled Trials
Source: J Med Internet Res. 2022 Jun 3;24(6):e30065. doi: 10.2196/30065 (PMC9206197; doi:10.2196/30065)

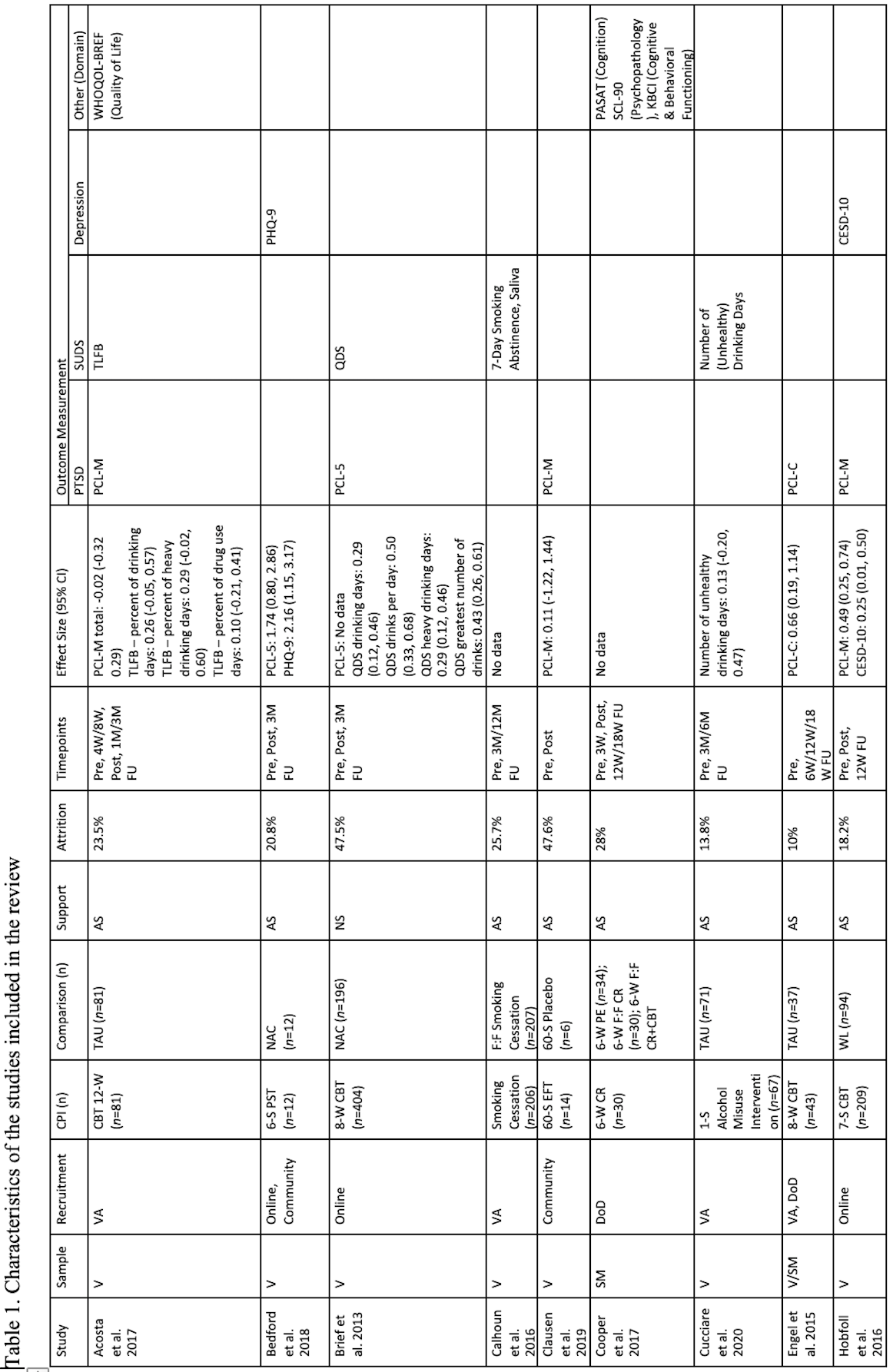


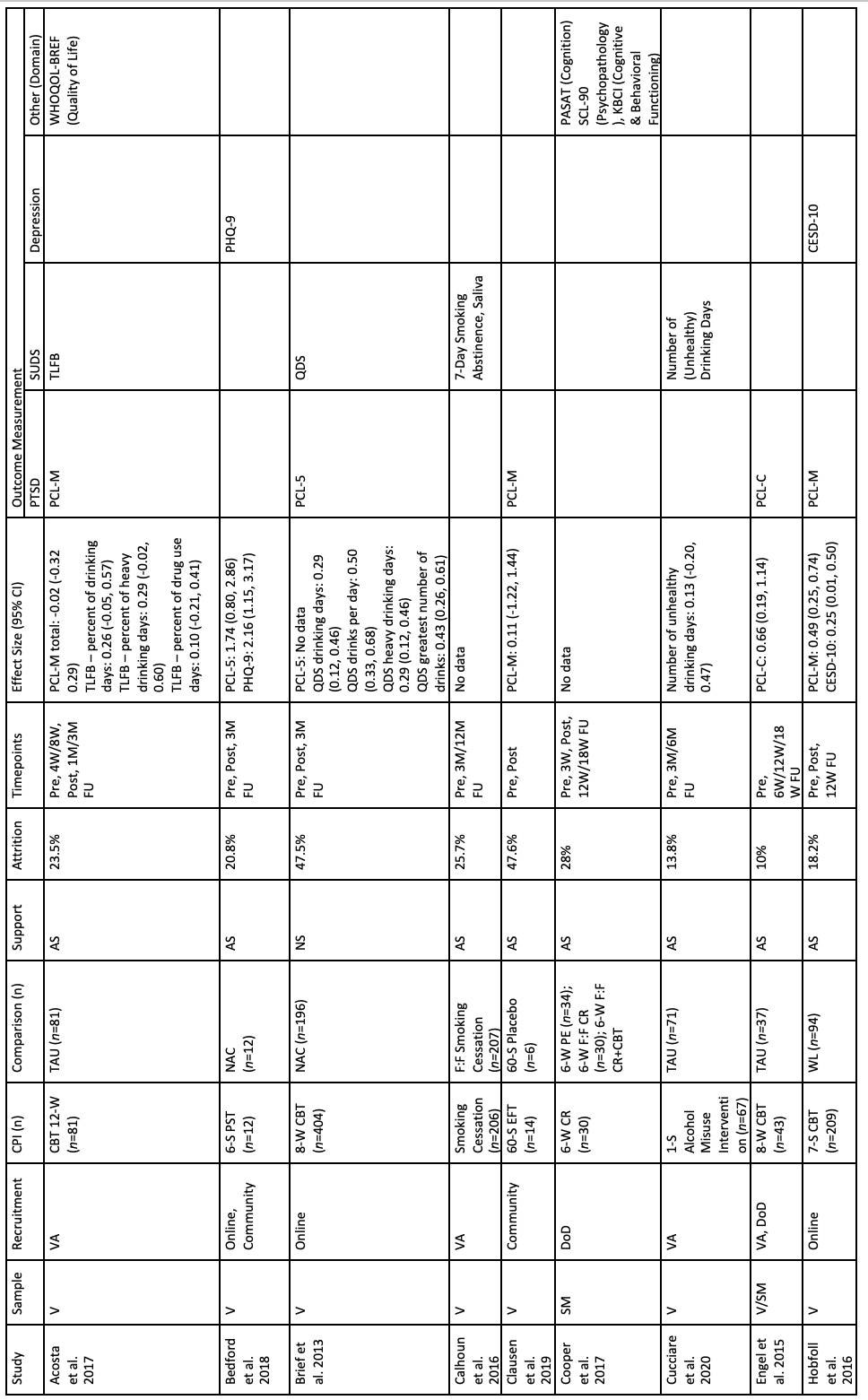


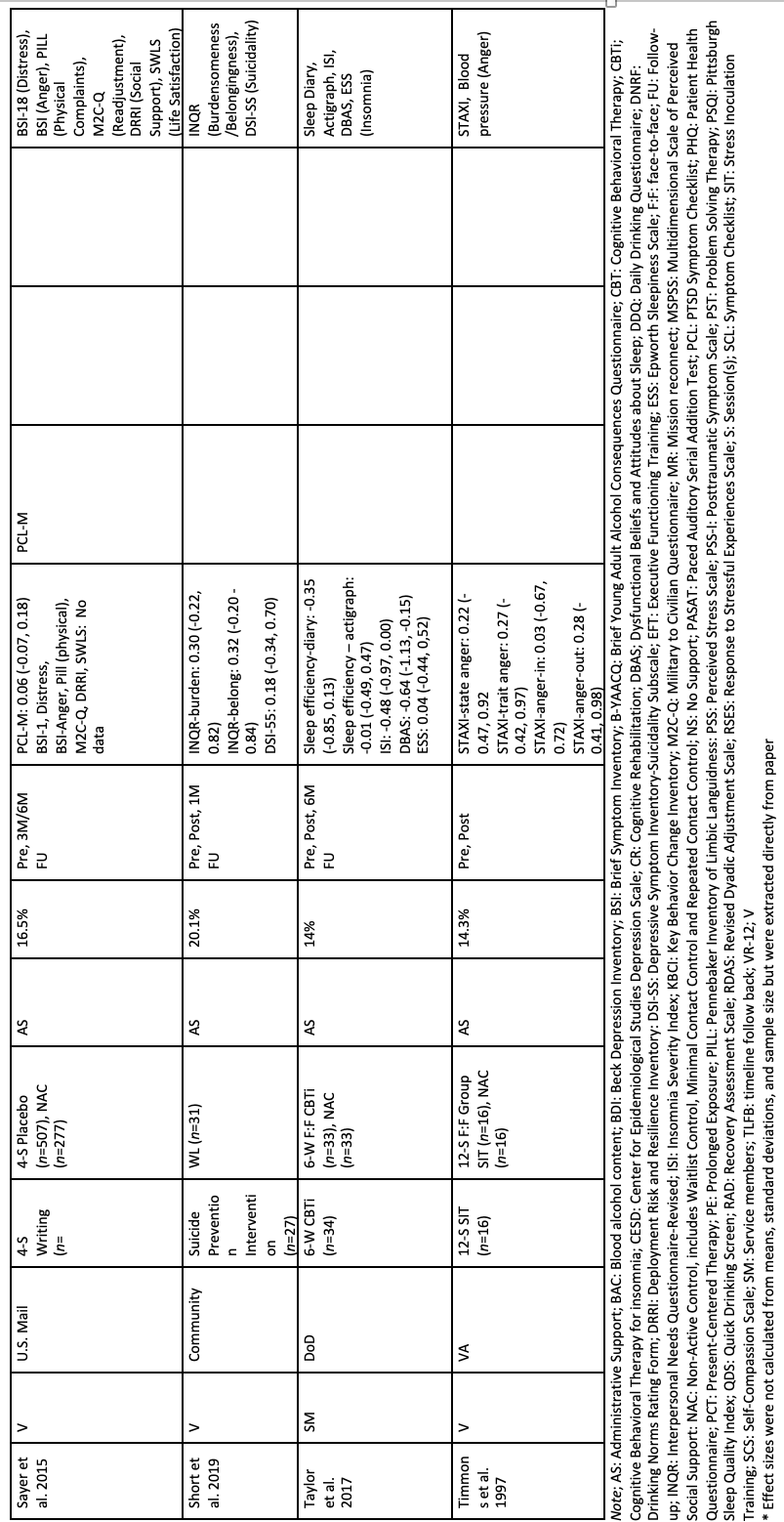

Supplement: Multimedia Appendix 2 [file jmir_v24i6e30065_app2.docx]
